# Construction of Three High-Density Genetic Linkage Maps and Dynamic QTL Mapping of Growth Traits in Yellow River Carp (*Cyprinus carpio haematopterus*)

**DOI:** 10.3390/cimb43030160

**Published:** 2021-12-17

**Authors:** Lei Wang, Songpeng Jia, Yuxuan Zhang, Shuhong Jiang, Yuhan Chen, Junping Chen, Miao Yu, Lan Zhang, Zhigang Qiao, Xuejun Li

**Affiliations:** 1 College of Fisheries, Henan Normal University, Xinxiang 453007, China; 18749298991@163.com (S.J.); Zhangyuxuan1004@163.com (Y.Z.); jsh2274187363@163.com (S.J.); c19115598868@163.com (Y.C.); chenjunpinghn@163.com (J.C.); miaoyu@htu.edu.cn (M.Y.); zhanglan_1216@163.com (L.Z.); 13503800008@126.com (Z.Q.); 2 Engineering Lab of Henan Province for Aquatic Animal Disease Control, Henan Normal University, Xinxiang 453007, China; 3 Engineering Technology Research Center of Henan Province for Aquatic Animal, Xinxiang 453007, China; 4 Changyuan Branch, Henan Academy of Agricultural Sciences, Changyuan 453400, China

**Keywords:** *Cyprinus carpio haematopterus*, modified GBS method, linkage map, dynamic QTL, growth-related trait

## Abstract

To provide the theoretical basis for researching growth, development, and molecular marker-assisted breeding of the economically important Yellow River carp (*Cyprinus carpio haematopterus*) using dynamic quantitative trait locus (QTL) mapping, we constructed three genetic linkage maps from 207 progeny using a new modified genotyping-by-sequencing method. The three maps contained 16,886, 16,548, and 7482 single nucleotide polymorphism markers, respectively, with an average interval of 0.36 cM, 0.45 cM, and 1.00 cM. We identified 148 QTLs related to four growth traits that were located on 25 chromosomes from three growth stages of Yellow River carp. A total of 32, 36, 43, and 37 QTLs were associated with body length, height, width, and weight, respectively. Among them, 47 QTLs were detected for only one growth trait in one stage, but all of the other QTLs were co-localized. Of the 14 main QTLs, 13 were located on chromosome 12, which suggests the presence of growth-related genes on this chromosome. We then detected 17 candidate genes within 50 K upstream and downstream of the 14 main QTLs. This is the first report of the dynamic QTL mapping of growth traits of Yellow River carp, and the results can be used in future studies of growth, development, and molecular-assisted breeding of this species.

## 1. Introduction

The construction of genetic linkage maps and quantitative trait locus (QTL) mapping are very important techniques for the study of fish genetics and breeding. Many genetic linkage maps have been constructed for common carp (*Cyprinus carpio*) since the first map was reported in 2000 [1]. In subsequent studies, markers such as simple sequence repeat (SSR) and single nucleotide polymorphism (SNP) were gradually applied to construct the genetic linkage map of common carp [2,3].

Yellow River carp (*Cyprinus carpio haematopterus*) is one of the most economically important aquatic fish in the world. It is a well-known endemic species of common carp in the Yellow River valley, and it is also an important freshwater cultured fish in China. This species is famous in China because of its golden scales and red tail, long body shape, and delicious tender meat. It also has strong cold resistance, a high feed conversion rate, and is easy to catch. Although there are some QTL mapping [4,5,6] and adaptive evolution [7] studies of Yellow River carp, there are only a few genetic linkage maps, and dynamic QTL mapping has not been reported.

Some scholars have conducted QTL mapping of the growth-related traits of common carp. For example, Laghari et al. selected 109 SSR markers, 31 expressed sequence tag SSR (EST-SSR) markers, and 167 SNP markers, and they identified 7 QTLs associated with body weight and growth rate [8]. With the completion of common carp genome sequencing [9] and the development of simplified genome sequencing technology, the density of the common carp genetic linkage map and the accuracy of QTL mapping are increasing. Peng et al. selected 28,194 SNP markers and detected 22 growth-related QTLs and 7 sex-related QTLs [5]. Chen et al. conducted QTL mapping and a genome-wide association analysis of the head type traits of Yellow River carp and identified 12 loci that were significantly related to head type [6]. Feng et al. mapped the growth traits of the F2 population of Yangtze River carp and found 21 growth-related QTLs that could explain the observed phenotypic variation (PVE) [10]. Wang et al. constructed a high-density genetic linkage map based on a common carp F2 family using a streamlined restriction site-associated DNA genotyping method based on sequencing the uniform fragments produced by type IIB restriction enzymes [4]. They then carried out QTL mapping studies and identified 12 chromosomal QTLs and 2 genome-wide growth-related QTLs.

Many QTL mapping studies of fish have focused on a certain stage of development, which is generally defined by the researcher based on the study question. For example, in aquaculture studies, QTL mapping of fish weight could be focused on the time when a fish grows to commercial size. This approach allows only a static study of the cumulative effect of these traits, and it cannot reflect the effect of genes on the whole growth process. To address this issue, Zhu proposed the conditional QTL and unconditional QTL analysis methods based on the composite interval mapping of a mixed linear model, which some scholars refer to as dynamic mapping [11]. Wu et al. developed software to locate the net genetic effect QTL from time “1” to “t” [12]. Other researchers have conducted dynamic QTL analyses of several species, including the potato (*Solanum tuberosum*) [13], wheat (*Triticum aestivum*) [14], rice (*Oryza sativa*) [15], and cotton (*Gossypium hirsutum*) [16].

Similar methods have been used for dynamic QTL mapping in fish. McClelland and Naish mapped 53 QTLs related to three traits by measuring the growth rate, body length, and body weight of coho salmon (*Oncorhynchus kisutch*) over eight time periods [17]. Laghari et al. studied the growth rate and body weight of 92 individuals of F1 common carp at 10, 11, and 12 months of age and identified 7 QTLs related to body weight [8]. Gutierrez et al. marked Atlantic salmon (*Salmo salar*) fry from five families with passive integrated transponders (PIT) when they reached 25 g, and then used SNP markers to locate the weight-related QTLs at four time points [18]. In the weight-related QTL detection of gilthead seabream (*Sparus aurata*) at four time points in a growth cycle, the QTLs of linkage group 1 were significant at all time points, but the effect varied with time, and one QTL of linkage group 21 seemed to affect the later growth weight of the species [19].

In 2018, Qi et al. [20] invented a modified genotyping-by-sequencing (GBS) method, which is a simplified genome sequencing technique based on GBS technology that is better at constructing polyploid linkage maps than previously available methods [21]. In the modified GBS, the size of the recovered fragment is adjusted by adjusting the volume ratio of the magnetic bead solution to the connecting product, which avoids uncontrollable processes, such as glue cutting and recovery, and improves the repeatability of the technology. Qi et al. also used a new analysis process to greatly improve genotyping accuracy [21]. The modified GBS is suitable for outcrossed polyploid species, complex genome species, and species with high repetitive sequences [20]. In this study, we will use the modified GBS and the new analysis process to analyze the experimental results. By comparing the QTLs associated with growth traits at different time periods, we may provide a theoretical basis for studying the growth and development mechanisms at work in Yellow River carp. Our results may also be applicable to the molecular marker-assisted breeding of Yellow River carp.

## 2. Materials and Methods

### 2.1. Mapping Family and Measuring Phenotype

In May 2017, one female fish was selected from a fast-growing Yellow River carp family established in 2015 and one male fish was selected from a slow-growing Yellow River carp family. These parent fish were allowed to mate to establish the F1 family. When the average body length of the progeny reached about 10 cm at 5 months old, 300 fish were randomly selected and injected with PIT markers. Their body length, height, thickness, and weight were measured at 13, and 17 months of age using an electronic balance (Wuxin Weighing Instrument Co., Ltd., Ningbo, China) and Vernier calipers (Ningbo Deli Co., Ltd., Ningbo, China). During the last measurement, a piece of the caudal fin of each fish was cut and preserved with anhydrous ethanol. After dead fish and those whose markers could not be detected were removed from the sample, 207 F1 progeny remained as the mapping population. Their genomic DNA was isolated from fins using the traditional proteinase-K digestion and phenol-chloroform extraction method. The quality of the genomic DNA was checked using a NanoDrop 2000 UV-vis spectrophotometer (Thermo Scientific, Waltham, MA, USA). The average value, standard deviation of each trait and Pearson correlation coefficient between traits were performed using SPSS24.0 (IBM Corp, Armonk, NY, USA).

### 2.2. GBS Sequencing and Creation of Maternal (HA), Paternal (AH), and Both Parent (HH) SNP Datasets

The DNA samples were sent to Shanghai Ouyi Biotechnology Co., Ltd. (Shanghai, China) for sequencing. The libraries of the parents and 207 F1 progeny were prepared following the methods of Qi et al. [21]. Read processing, SNP calling and filtering were conducted following the methods of Qi et al. [21]. Chi-square tests were used to determine the markers’ deviation from the expected Mendelian segregation ratios. Highly distorted markers (*p*-value ≤ 1 × 10^−10^) were discarded. The remaining SNPs were categorized according to their segregation ratio and parental genotype. Population type was set as “backcross” (BC) for the maternal and paternal data sets, and as “F2 intercross” for the HH data set [21]. SNPs that were heterozygous in both parents with progeny segregating as A:H:B (1:2:1) constituted the HH dataset. SNPs with genotypic score A or B in parent 1 and H in parent 2 and segregating as A:H or B:H (1:1) were combined, and the B scores were converted to A scores to form the paternal (AH) dataset. Similarly, the H:A and H:B markers were combined, and the B scores were converted to A scores to yield the maternal (HA) dataset, following the methods of Qi et al. [21]. The GBS reads were submitted to NCBI-SRA (Acc. PRJNA788161).

### 2.3. Map Construction and Estimation of Genome Size

The HA, AH, and HH genetic maps were constructed using a combination of MSTMap [22] and MAPMAKER (modified from Lander et al. [23] as described by Qi et al. [20]. The population type was set as “backcross” for the maternal and paternal datasets and as “F2 intercross” for the HH dataset. Co-segregating SNPs were added to the framework map to the same location as their representative marker using an in-house Python script. Linkage maps were drawn with MapChart [24].

### 2.4. QTL Location and Consensus QTL Analysis

The body length, height, thickness, and weight measured for the F1 progeny were used as inputs for the QTL analysis. QTL mapping was conducted with WinQTLCart 2.5 [25] using the composite interval mapping model. The population type for QTL analysis was set as “SF2” for the HH maps and as “B2” for the maternal and paternal maps. A walk speed of 0.5 cM was used for QTL identification. The logarithm of odds (LOD) threshold for significant QTLs (*p* ≤ 0.05) was determined by 1000 permutations. After the QTLs were located, all QTLs located within 1M base pairs of each other on the physical map were identified as being consensus QTLs.

### 2.5. Screening for Growth-Related Candidate Genes

Potential growth-related candidate genes within QTLs with LOD value > 5.5 and PVE > 10% were identified. Briefly, we screened the +50 kb genome regions surrounding the significant SNPs based on the common carp reference genome (BIGD Genome Warehouse, https://bigd.big.ac.cn/gwh/Assembly/497/show (accessed on 9 May 2019) and annotated the candidate genes by conducting BLAST analysis against the Swiss-Prot database.

## 3. Results

### 3.1. Phenotype Data

It can be seen from Table 1 that the body weight in the T1T2 period (4 months) accounted for more than 66% of the body weight in the T2 period (17 months). Pearson correlation analysis showed that there is a significant correlation between each trait at the 0.01 level (Table 2). However, the correlation coefficients between the traits differed greatly, as the smallest was only 0.348, and the largest was 0.987. The correlation coefficients of BL, BH and BT in the T1T2 stage for all traits were <0.8, and the correlation coefficients among all other traits were >0.8. These results show that the increase of body mass in the T1T2 stage is caused by many traits, rather than being caused mainly by a certain trait.

### 3.2. GBS Tags

High-throughput sequencing of the 209 GBS libraries from the 2 parents and 207 progeny yielded 6.9 and 7.2 million clean reads for the dam and sire, respectively, and 1.1 billion reads for the offspring, with an average of 5.6 million reads per progeny. The sequencing values were converted into genotypes, and only the recombination events across the progeny were retained for linkage map construction. The simplified linkage map (one marker per 2 cM) was used for the permutation calculation. Finally, 16,886 SNPs that were heterozygous only in the female parent were used to construct the maternal map, 16,548 SNPs that were heterozygous only in the male parent were used to construct the paternal map, and 7482 SNPs that were heterozygous in both the male and female parents were used to construct the HH map.

### 3.3. Construction of the Genetic Linkage Map

**Figure 1:**
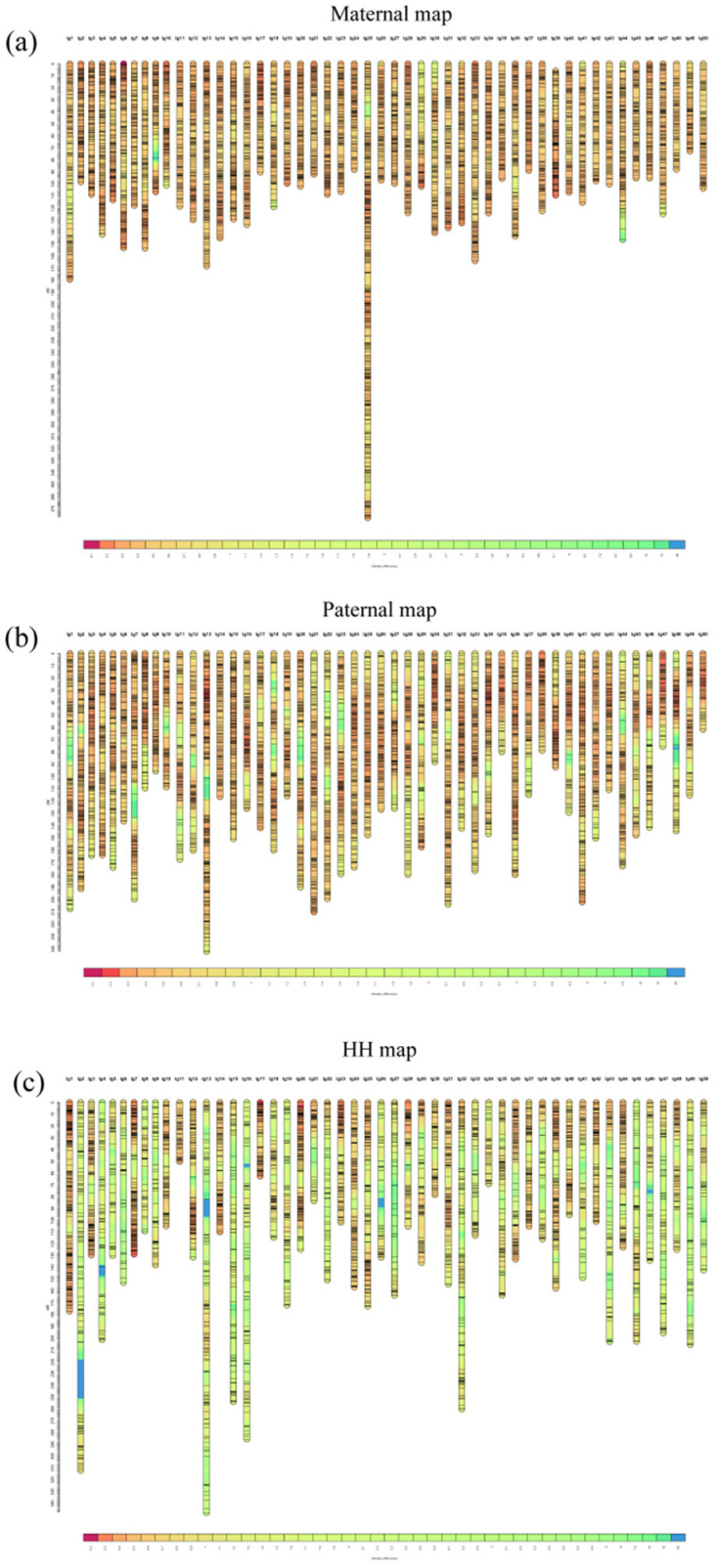
High-density genetic linkage maps of maternal (**a**), paternal (**b**), and HH (**c**) Yellow River carp.

Three genetic linkage maps (maternal, paternal, and HH) that included 50 linkage groups were constructed (Figure 1). The maternal map contained 16,886 SNPs and spanned 6103.6 cM, with an average marker interval of 0.36 cM (Table S1, see Supplementary Materials). The number of markers per group varied from 183 (linkage group 49, LG49) to 881 (LG25), with an average of 337.7 cM, and the lengths of the LGs ranged from 72.6 cM (LG49) to 377.5 cM (LG25), with an average of 122.1 cM. The paternal map consisted of 16,548 SNPs and spanned 7370 cM, with an average marker interval of 0.45 cM. The number of markers in each group varied from 201 (LG50) to 626 (LG13), with an average of 331, and the lengths of the LGs ranged from 72.6 cM (LG49) to 377.5 cM (LG25), with an average of 122.1 cM. The HH map consisted of 7480 SNPs and spanned 7454.9 cM, with an average marker interval of 1.00 cM. The number of markers in each group varied from 70 (LG1) to 401 (LG 34), with an average of 149.6, and the lengths of the LGs ranged from 50 cM (LG11) to 347.8 cM (LG13), with an average of 149.1 cM (Table S1).

### 3.4. QTLs for Growth Traits

**Figure 2:**
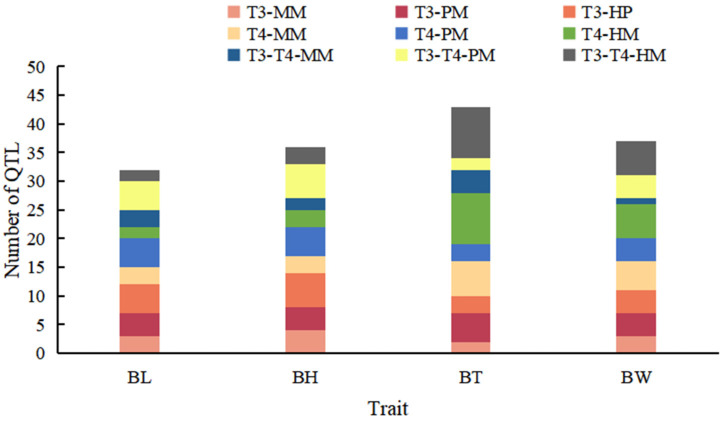
Distribution of QTLs for body length (BL), body height (BH), body thickness (BT), and body weight (BW) detected at three stages; MM, maternal map; PM, paternal map; HM, HH map; T1, 13 months after hatch (mah); T2, 17 mah; T1T2, 13–17 mah.

QTL fine mapping based on the high-density genetic linkage maps identified 148 QTLs, 8 of which were not located on chromosomes (Table S2). Thirty-two QTLs associated with body length were distributed in 11 LGs and 8 chromosomes (Figure S1), with logarithm of odds (LOD) scores ranging from 3.01 to 13.20 and PVEs ranging from 0.10% to 13.20%. The 36 QTLs associated with body height were distributed in 14 LGs and 11 chromosomes (Figure S2), with LOD scores ranging from 3.06 to 7.91 and PVEs ranging from 0.38% to 12.43%. Forty-three QTLs associated with body thickness were distributed in 18 LGs and 17 chromosomes (Figure S3), with LOD scores ranging from 3.00 to 7.60 and PVEs ranging from 0.01% to 12.20%. The 37 QTLs associated with body weight were distributed in 12 LGs and 10 chromosomes (Figure S4), with LOD scores ranging from 3.01 to 6.97 and PVEs ranging from 2.26% to 13.06%. Table S2 and Figure 2 show that there were great differences in the number and positions of QTLs for different traits in different stages, and some QTLs were expressed only during a specific time period.

### 3.5. Analysis of Consensus QTLs (Cqtls)

In total, 101 QTLs were integrated into 26 consensus Cqtls (Table 3), and 47 QTLs were detected for only one growth character in one stage. These Cqtls covered 18 linkage groups and 15 chromosomes, and had LODs ranging from 3.00 to 7.95 and PVEs ranging from 0.01% to 13.20%. Fourteen Cqtls contained 2 QTLs, 5 Cqtls contained 3 QTLs, 3 Cqtls contained 4 QTLs, 2 Cqtls contained 6 QTLs, 1 Cqtls contained 7 QTLs, and 1 Cqtl contained 27 QTLs. Among the 26 Cqtls, 14 Cqtls were detected only for one trait. Cqtl-6 and Cqtl-25 were detected only at T1, and Cqtl-26 was detected only at T2. The other nine Cqtls were detected for multiple traits and time periods.

### 3.6. Candidate Growth-Related Genes

After BLAST searching against the genome of Yellow River carp using extended SNP marker sequences, 17 potential candidate genes were identified based on the SNP position in the genome (Table 4). The UPF0609 protein C4orf27 homolog (*cd027*), ankyrin-2 (*ank2*), and calcium/calmodulin-dependent protein kinase type II delta (*camk2d*) genes located on chromosome 1 were associated with body height at stage T1T2, and the other 14 candidate genes were located on chromosome 12. Synaptotagmin-1 (*syt1*) was associated with five QTLs covering BW, BT, BH and three stages. Zinc finger NFX1-type containing 1 (*znfx1*) was associated with body height at stage T1. Small nuclear ribonucleoprotein-associated protein B′ (*snrpb*) was associated with six QTLs covering all four traits and three stages. Itchy E3 ubiquitin protein ligase (*itch*) was associated with five QTLs covering BH, BT, BW and three stages. N-terminal EF-hand calcium-binding protein 1 (*neca1*) and protein CBFA2T2 (*cbfa2t2*) were associated with five QTLs covering all four traits and three stages. The remaining eight candidate genes were associated with five QTLs covering BH, BT, and BW at different times. Most of these candidate genes were related to cell proliferation, energy metabolism, immunity, and growth.

## 4. Discussion

High-density genetic linkage maps can be used for QTL fine mapping, which is helpful for identifying candidate genes associated with growth-related traits [26,27]. Simplified genome sequencing technology has become one of the main methods of constructing high-density genetic linkage maps [28,29,30,31,32], and researchers have used it to construct maps for aquatic animals such as pikeperch (*Sander lucioperca*) [33], large yellow croaker (*Larimichthys crocea*) [34], Ussuri catfish (*Pseudobagrus ussuriensis*) [35], and redtail catfish (*Hemibagrus wyckioides*) [36].

The modified GBS method is a simplified genome sequencing technique based on GBS technology that is better at constructing polyploid linkage maps than previously available methods [20,21]. Using this technique, we constructed three independent genetic linkage maps of Yellow River carp. Prior to our study (Table S3), the largest sample size of carp used to create a linkage map was 190 (627 markers) [37], and the highest density was 0.38 cM [5]. Thus, the mapping population used in this study (207 progeny) is the largest to date, and the map density is the highest to date. We constructed three independent genetic linkage maps because (i) we used the F1 population, (ii) the construction methods for the different markers differed, and (iii) the three maps were more in line with the genetic characteristics of the mapping population. The population type was set as “backcross” for the maternal and paternal datasets and as “F2 intercross” for the HH dataset. The mapping method used in this paper is different from the previous mapping methods [4,5], and the map made by this new method provides a new choice for the QTL location of Yellow River carp.

Dynamic QTL mapping can detect the expression of trait-related genes in different growth stages. We detected 148 QTLs located on chromosomes in different growth periods, and 47 of them were only detected for a certain trait in a certain time period. Seven QTLs were detected for different traits in the same time period, 37 QTLs were detected in two time periods, and 57 QTLs were detected in three time periods. These results show that some genes are continuously expressed throughout the growth and development period, whereas others are only expressed at a certain stage of development. This is consistent with the results of the correlation analysis of growth traits, which show that there is a certain correlation among the four traits in the three periods. Among them, the correlation between body length, body height, body width and the other traits in the T1T2 period was relatively low (0.3–0.8), while the correlation coefficients among other traits were >0.8. Gutierrez et al. reported similar results for Atlantic salmon. They analyzed the QTLs associated with net weight gain between time periods and found that the QTLs associated with weight appeared in different time periods in different families [18]. Similar results were found in the analysis of weight-related QTLs at four time points in one growth cycle of gilthead seabream. Some QTLs can be detected in multiple periods, while others can only be detected in a certain period [19]. We detected 47 QTLs in the T1T2 period of Yellow River carp growth, and 22 of them only existed in this growth stage. Most of the QTLs were different, so we then located conditional QTLs that were associated with growth traits. Compared with previous research about the growth-related QTL mapping of Yellow River carp only for a specific time [4,5], in this study we located the growth-related QTLs in three different time periods, which included not only two specific time points, but also the growth traits in a specific time period. This approach allowed us to explore the changes to the growth-related QTLs of the Yellow River carp.

Although conditional QTL mapping is rarely used in aquatic animals, it can play a very important role in breeding. For example, after injecting fish with PIT tags, their body mass can be measured before and after winter and then the QTLs associated with cold tolerance can be mapped [38]. The early growth traits of fish are greatly affected by motherhood [39,40], and dynamic QTL mapping can reduce the influence of the maternal effect and locate growth-related QTLs more exactly. Additionally, different types of fish have different growth characteristics, and measuring growth traits from the early growth stages to adulthood allows for more accurate QTL mapping. Conditional QTL can also be used to study gonadal development by detecting the condition before and after gonadal development in fish [41].

Many QTLs exhibit pleiotropy, meaning that a QTL is associated with different traits. Sometimes, one gene may affect multiple growth traits at the same time. For example, Cqtl-7, Cqtl-19, and Cqtl-21 in this study were detected for different traits in all three time periods. Similar results were reported previously for barley (*Hordeum vulgare*) [42] and Yellow River carp [4]. Through our QTL mapping analysis of the growth traits of Yellow River carp, we found that the QTL distribution showed a regionalization trend, with some growth traits having co-located QTLs. The QTLs with LOD > 5.5 and PVE > 10% were localized on chromosome 1 (BH-MM-T1T2-2) and chromosome 12 (the other 14 QTLs), which indicated that the major genes related to growth were localized on chromosomes 1 and 12. However, these results differed from those reported by Wang et al. [4] and Peng et al. [5], possibly due to the different genetic backgrounds of the families in the different studies. We also identified 47 QTLs associated with growth traits that were detected only in a single time period and for a single trait, which indicated that these QTLs were greatly affected by the growth period and environment. The LOD and PVE values of these QTLs were relatively small, which suggested that the QTLs detected in only one period and trait had little effect on growth traits.

In this study, the QTLs with LOD value > 5.5 and PVE > 10% were mainly concentrated on chromosome 12 and contained 14 candidate genes. Most of these genes are associated with cell proliferation, energy metabolism, immunity, and growth, but there may also be some as-yet unidentified functions. In future studies we will explore the relationship between alleles of these genes and growth, and we will screen for a number of molecular markers and alleles closely related to growth in order to provide a basis for the molecular marker-assisted selection of Yellow River carp.

## Figures and Tables

**Table 1:** Full length, body height, body thickness and body weight of Yellow River carp at the T1, T2 and T1T2 stages.

Trait	T1	T2	T1T2
Full length/cm	14.3 ± 3.5	20.6 ± 4.5	6.4 ± 1.6
Body height/cm	4.4 ± 1.0	6.2 ± 1.4	1.8 ± 0.6
Body thickness/cm	2.5 ± 0.6	3.7 ± 0.9	1.3 ± 0.4
Body weight/g	82.2 ± 56.8	243.1 ± 155.4	161.7 ± 102.5

**Table 2:** Pearson correlations between the traits in Yellow River carp at the T1, T2 and T1T2 stages.

	T1-BL	T1-BH	T1-BT	T1-BW	T2-BL	T2-BH	T2-BT	T2-BW	T1T2-BL	T1T2-BH	T1T2-BT	T1T2-BW
T1-BL	1	0.946 **	0.948 **	0.964 **	0.945 **	0.923 **	0.894 **	0.929 **	0.445 **	0.537 **	0.441 **	0.875 **
T1-BH		1	0.943 **	0.943 **	0.912 **	0.922 **	0.881 **	0.913 **	0.471 **	0.442 **	0.424 **	0.862 **
T1-BT			1	0.941 **	0.909 **	0.912 **	0.884 **	0.899 **	0.457 **	0.516 **	0.348 **	0.842 **
T1-BW				1	0.914 **	0.911 **	0.885 **	0.956 **	0.439 **	0.513 **	0.434 **	0.895 **
T2-BL					1	0.951 **	0.909 **	0.953 **	0.713 **	0.657 **	0.527 **	0.938 **
T2-BH						1	0.918 **	0.937 **	0.625 **	0.755 **	0.541 **	0.916 **
T2-BT							1	0.903 **	0.573 **	0.633 **	0.746 **	0.878 **
T2-BW								1	0.618 **	0.624 **	0.529 **	0.987 **
T1T2-BL									1	0.649 **	0.499 **	0.693 **
T1T2-BH										1	0.535 **	0.663 **
T1T2-BT											1	0.562 **
T1T2-BW												1

Note: ** indicates that the correlation is significant at the 0.01 level (2-tailed); T1, T2 and T1T2 indicate 13 months after hatch (mah), 17 mah and 13–17 mah respectively; BL, BH, BT and BW indicate body length, body height, body thickness and body weight, respectively.

**Table 3:** Consensus QTLs identified for growth traits.

Consencus QTL	QTL	LOD	PVE (%)	Marker Position	Marker Chromosome	No. of QTLs
Cqtl-1	BH-MM-T1T2-2	6.33	11.69	24,895,873	1	2
	BL-MM-T2-1	3.42	5.78			
Cqtl-2	BT-HM-T2-2	3.22	4.20	29,195,166	1	2
	BT-HM-T1T2-2	3.22	4.20			
Cqtl-3	BT-HM-T2-1	3.09	0.67	1,774,640	3	2
	BT-HM-T1T2-1	3.09	0.67			
Cqtl-4	BT-HM-T1-1	3.31	3.13	17,045,253	4	3
	BT-HM-T2-3	3.15	0.26			
	BT-HM-T1T2-3	3.15	0.26			
Cqtl-5	BL-HM-T2-2	4.42	4.15	22,794,002	12	3
	BL-HM-T1T2-2	4.42	4.15			
	BW-PM-T2-1	5.52	9.34	23,575,083		
Cqtl-6	BL-PM-T1-1	7.95	13.20	24,258,680	12	2
	BT-PM-T1-1	7.60	12.20			
Cqtl-7	BH-PM-T2-1	7.02	11.70	24,883,274	12	27
	BL-PM-T1-2	6.79	11.43	25,754,793		
	BL-PM-T2-1	6.11	9.61	24,883,274		
	BL-PM-T2-2	5.63	8.90	25,754,793		
	BL-PM-T1T2-3	5.34	9.24	24,980,556		
	BL-HM-T1-1	4.60	6.91	25,680,671		
	BL-HM-T2-1	3.93	2.78	25,507,539		
	BL-HM-T1T2-1	3.93	2.78			
	BH-PM-T1-1	7.91	12.43	24,883,274		
	BH-PM-T1-2	7.18	11.38	25,754,793		
	BH-PM-T2-2	6.65	11.13			
	BH-HM-T1-2	5.89	11.20	25,680,671		
	BH-HM-T2-2	3.18	0.62	25,735,909		
	BH-HM-T1T2-2	3.18	0.62			
	BT-PM-T1-2	6.62	10.75	25,754,793		
	BT-PM-T2-2	6.16	10.43	24,883,274		
	BT-PM-T2-3	5.31	9.07	25,754,793		
	BT-HM-T1-2	3.79	7.26	25,680,671		
	BW-PM-T1-1	5.70	9.92	24,883,274		
	BW-PM-T1-2	4.86	8.55	25,754,793		
	BW-PM-T2-2	6.77	11.30	24,883,274		
	BW-PM-T2-3	5.64	9.54	25,754,793		
	BW-PM-T1T2-1	6.97	13.06	24,883,274		
	BW-PM-T1T2-2	6.53	12.28	25,754,793		
	BW-HM-T1-1	5.58	5.00	25,680,671		
	BW-HM-T2-2	5.33	4.77			
	BW-HM-T1T2-2	5.57	5.11			
Cqtl-8	BH-PM-T1T2-5	3.59	5.95	33,896,198	13	2
	BH-HM-T1-3	4.70	6.87	33,051,888		
Cqtl-9	BH-HM-T2-1	3.60	5.24	6,651,371	17	2
	BH-HM-T1T2-1	3.60	5.24			
Cqtl-10	BT-HM-T2-6	3.12	0.01	9,979,524	18	2
	BT-HM-T1T2-6	3.12	0.01			
Cqtl-11	BT-HM-T2-4	3.80	0.13	2,672,269	19	2
	BT-HM-T1T2-4	3.80	0.13			
Cqtl-12	BT-HM-T2-5	3.71	0.10	6,033,807	19	2
	BT-HM-T1T2-5	3.71	0.10			
Cqtl-13	BT-HM-T2-7	3.22	1.61	8,855,465	28	2
	BT-HM-T1T2-7	3.22	1.61			
Cqtl-14	BW-HM-T1-2	3.03	4.90	14,096,508	29	3
	BW-HM-T2-3	3.06	4.99			
	BW-HM-T1T2-4	3.04	4.87			
Cqtl-15	BH-HM-T2-3	3.30	6.27	18,356,606	29	4
	BH-HM-T1T2-3	3.30	6.27			
	BW-HM-T2-4	3.02	4.78	18,202,561		
	BH-HM-T1-5	3.14	1.83	19,199,891		
Cqtl-16	BL-MM-T1-3	4.77	8.32	5,507,998	34	2
	BT-MM-T2-3	4.76	8.37	6,379,096		
Cqtl-17	BT-HM-T2-8	3.02	1.32	10,669,401	34	2
	BT-HM-T1T2-8	3.02	1.32			
Cqtl-18	BW-HM-T1-3	3.42	3.20	4,996,409	39	3
	BW-HM-T2-5	3.33	3.10			
	BW-HM-T1T2-5	3.58	3.44			
Cqtl-19	BL-PM-T2-3	3.92	6.06	9,244,863	39	6
	BL-HM-T1-5	3.57	0.10	9,548,459		
	BH-PM-T1-3	3.88	5.85	8,819,887		
	BH-PM-T2-3	3.72	5.70			
	BT-PM-T1-3	3.00	4.69	9,244,863		
	BW-PM-T1T2-3	4.36	6.43	8,819,887		
Cqtl-20	BL-PM-T1-3	4.78	7.34	10,941,579	39	4
	BL-PM-T2-4	4.44	6.83	11,297,467		
	BH-PM-T2-4	4.12	6.28			
	BW-PM-T1-4	3.56	5.29	10,941,579		
Cqtl-21	BL-PM-T1-4	4.69	7.21	16,555,037	39	7
	BL-PM-T2-5	4.00	6.18			
	BL-HM-T1-4	3.58	0.01	16,165,443		
	BH-PM-T1-4	4.01	6.04	16,555,037		
	BH-PM-T2-5	3.91	5.98			
	BW-PM-T1T2-4	3.88	5.76			
	BT-PM-T1-4	3.28	5.10			
Cqtl-22	BW-PM-T2-4	3.17	4.61	19,390,144	39	4
	BW-HM-T1-4	3.69	4.17	18,795,619		
	BW-HM-T2-6	3.62	3.98			
	BW-HM-T1T2-6	3.71	4.29			
Cqtl-23	BT-HM-T2-9	3.07	2.48	12,218,984	40	2
	BT-HM-T1T2-9	3.07	2.48			
Cqtl-24	BH-MM-T1-4	3.67	6.43	2,458,218	44	6
	BH-MM-T2-3	4.08	7.15			
	BT-MM-T2-6	3.82	6.55			
	BT-MM-T1-2	4.09	7.37	3,318,682		
	BW-MM-T1-3	3.63	6.37			
	BW-MM-T2-3	3.01	5.81			
Cqtl-25	BT-MM-T1-1	4.41	7.92	5,304,386	44	2
	BW-MM-T1-2	3.46	6.09			
Cqtl-26	BW-MM-T2-4	3.01	5.16	17,239,666	50	3
	BW-MM-T2-5	3.21	5.49	16,482,578		
	BL-MM-T2-3	3.15	5.24	16,055,240		

Note: T1, T2 and T1T2 indicate 13 months after hatch (mah), 17 mah and 13–17 mah respectively; BL, BH, BT, BW indicate body length, body height, body thickness and body weight, respectively.

**Table 4:** Summary of the growth trait QTLs and candidate genes in Yellow River carp.

Number	Gene Name	Annotation	Gene_Start	Gene_End	Chr	QTL
1	*ank2*	Ankyrin-2	24,866,644	24,926,743	LG1	BH-MM-T1T2-2
2	*cd027*	UPF0609 protein C4orf27 homolog	24,860,171	24,864,690		
3	*camk2d*	Calcium/calmodulin-dependent protein kinase type II delta	24,930,027	24,966,141		
4	*syt1*	Synaptotagmin-1	24,858,871	24,869,140	LG12	BW-PM-T1T2-1; BW-PM-T2-2; BT-PM-T2-2; BH-PM-T2-1; BH-PM-T1-1
5	*manbl*	Protein MANBAL	24,873,472	24,874,795	LG12	BH-PM-T1-1; BH-PM-T2-1; BT-PM-T2-2; BW-PM-T2-2; BW-PM-T1T2-1
6	*nfatc2*	Nuclear factor of activated T-cells, cytoplasmic 2	24,876,144	24,887,937	LG12	
7	*rnf182*	E3 ubiquitin-protein ligase RNF182	24,897,900	24,898,634	LG12	
8	*rgs9bp*	Regulator of G-protein signaling 9-binding protein	24,900,978	24,901,894	LG12	
9	*ube2c*	Ubiquitin-conjugating enzyme E2 C	24,902,429	24,904,315	LG12	
10	*dnttip1*	Deoxynucleotidyltransferase terminal-interacting protein 1	24,905,995	24,909,672	LG12	
11	*pcif1*	Phosphorylated CTD-interacting factor 1	24,913,041	24,921,314	LG12	
12	*pltp*	Phospholipid transfer protein	24,928,567	24,932,818	LG12	
13	*znfx1*	Zinc finger NFX1-type containing 1	25,678,811	25,690,871	LG12	BH-HM-T1-2
14	*snrpb*	Small nuclear ribonucleoprotein-associated protein B′	25,708,066	25,711,568	LG12	BL-PM-T1-2; BH-PM-T1-2; BH-PM-T2-2; BH-HM-T1-2; BT-PM-T1-2; BW-PM-T1T2-2
15	*itch*	Itchy E3 ubiquitin protein ligase	25,714,170	25,726,476	LG12	BH-PM-T1-2; BH-PM-T2-2; BH-HM-T1-2; BT-PM-T1-2; BW-PM-T1T2-2
16	*neca1*	N-terminal EF-hand calcium-binding protein 1	25,732,452	25,764,815	LG12	BL-PM-T1-2; BH-PM-T1-2; BH-PM-T2-2; BT-PM-T1-2; BW-PM-T1T2-2
17	*cbfa2t2*	Protein CBFA2T2	25,771,196	25,808,027	LG12	

Note: T1, T2 and T1T2 indicate 13 months after hatch (mah), 17 mah and 13–17 mah respectively; BL, BH, BT and BW indicate body length, body height, body thickness and body weight, respectively.

## Data Availability

GBS reads were submitted to NCBI-SRA (Acc. PRJNA788161). Details about the genetic linkage maps are listed in Table S4.

## Supplementary Materials

The following are available online at https://www.mdpi.com/article/10.3390/cimb43030160/s1.
