# Supplementary material for: Construction of Three High-Density Genetic Linkage Maps and Dynamic QTL Mapping of Growth Traits in Yellow River Carp (Cyprinus carpio haematopterus)
Source: Curr Issues Mol Biol. 2021 Dec 17;43(3):2276–88. doi: 10.3390/cimb43030160 (PMC8928983; doi:10.3390/cimb43030160)
Supplement: Supplementary file 1 [file cimb-43-00160-s001.zip › Figure S3.pdf]

a

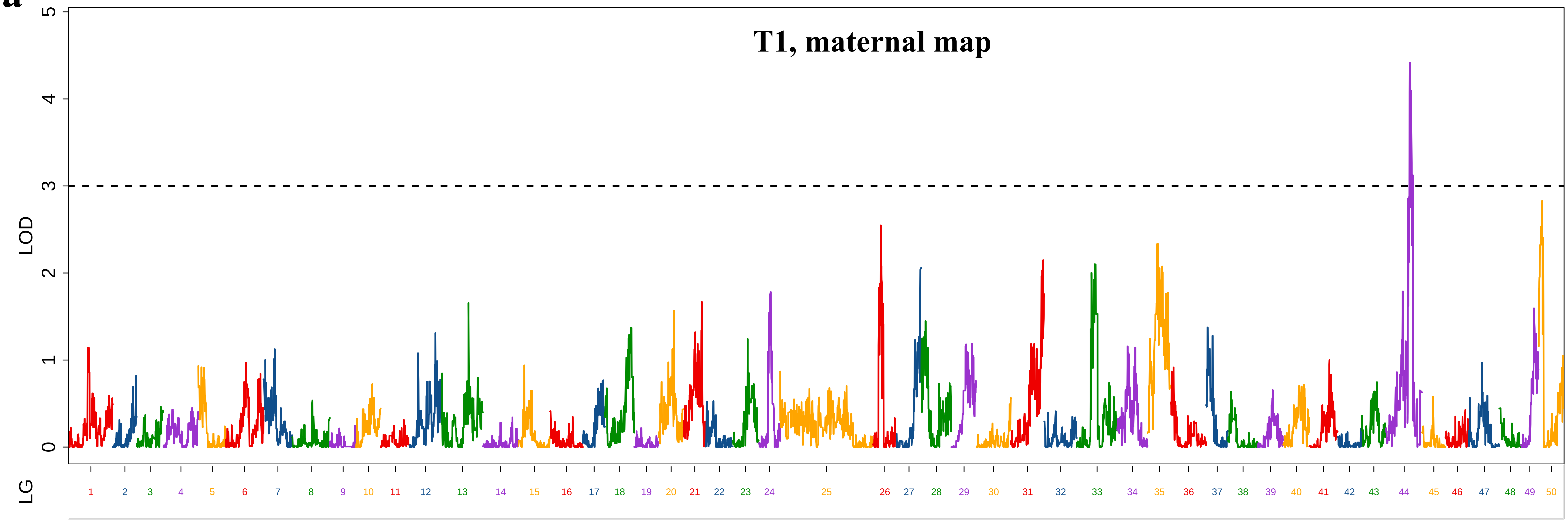

T1, maternal map
LOD
LG
0
1
2
3
4
5
1
2
3
4
5
6
7
8
9
10
11
12
13
14
15
16
17
18
19
20
21
22
23
24
25
26
27
28
29
30
31
32
33
34
35
36
37
38
39
40
41
42
43
44
45
46
47
48
49
50

b

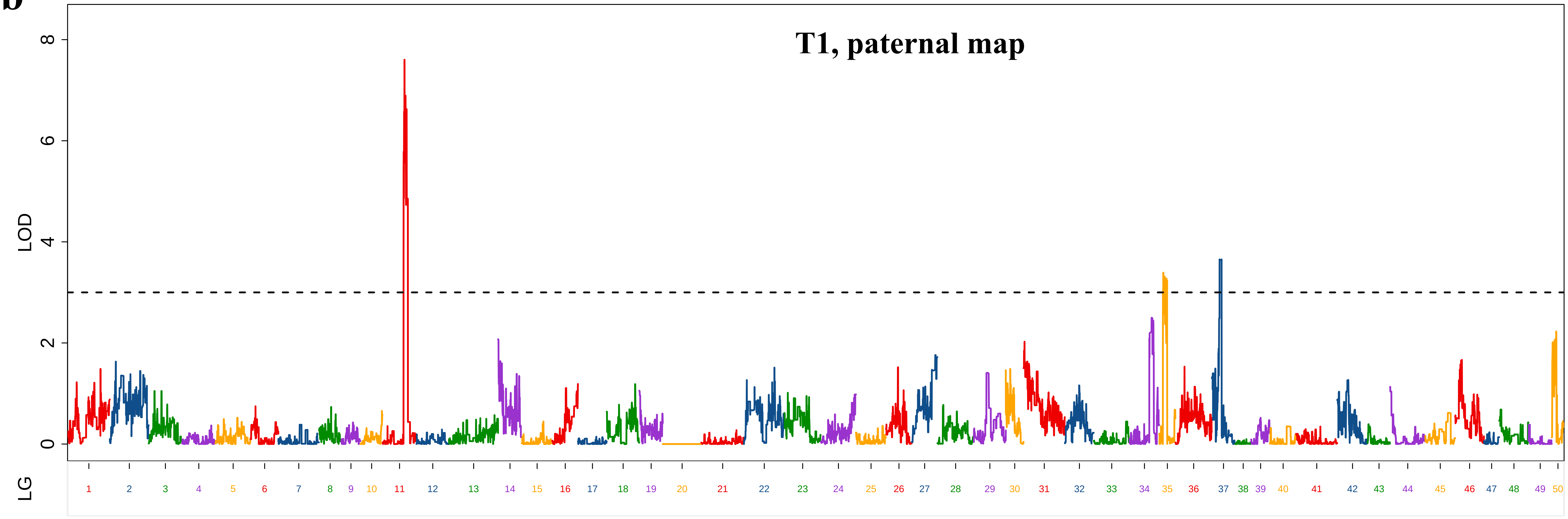
T1, paternal map
LOD
0
2
4
6
8
LG
1 2 3 4 5 6 7 8 9 10 11 12 13 14 15 16 17 18 19 20 21 22 23 24 25 26 27 28 29 30 31 32 33 34 35 36 37 38 39 40 41 42 43 44 45 46 47 48 49 50

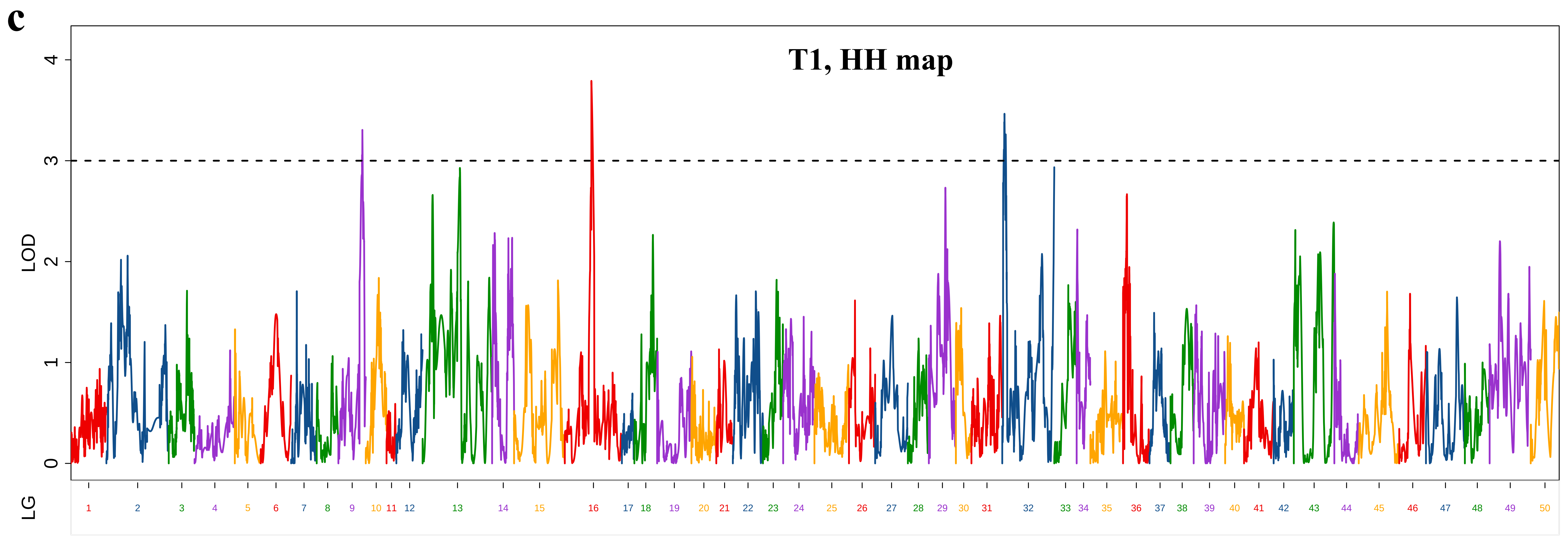
c
T1, HH map
LOD
0
1
2
3
4
LG
1 2 3 4 5 6 7 8 9 10 11 12 13 14 15 16 17 18 19 20 21 22 23 24 25 26 27 28 29 30 31 32 33 34 35 36 37 38 39 40 41 42 43 44 45 46 47 48 49 50

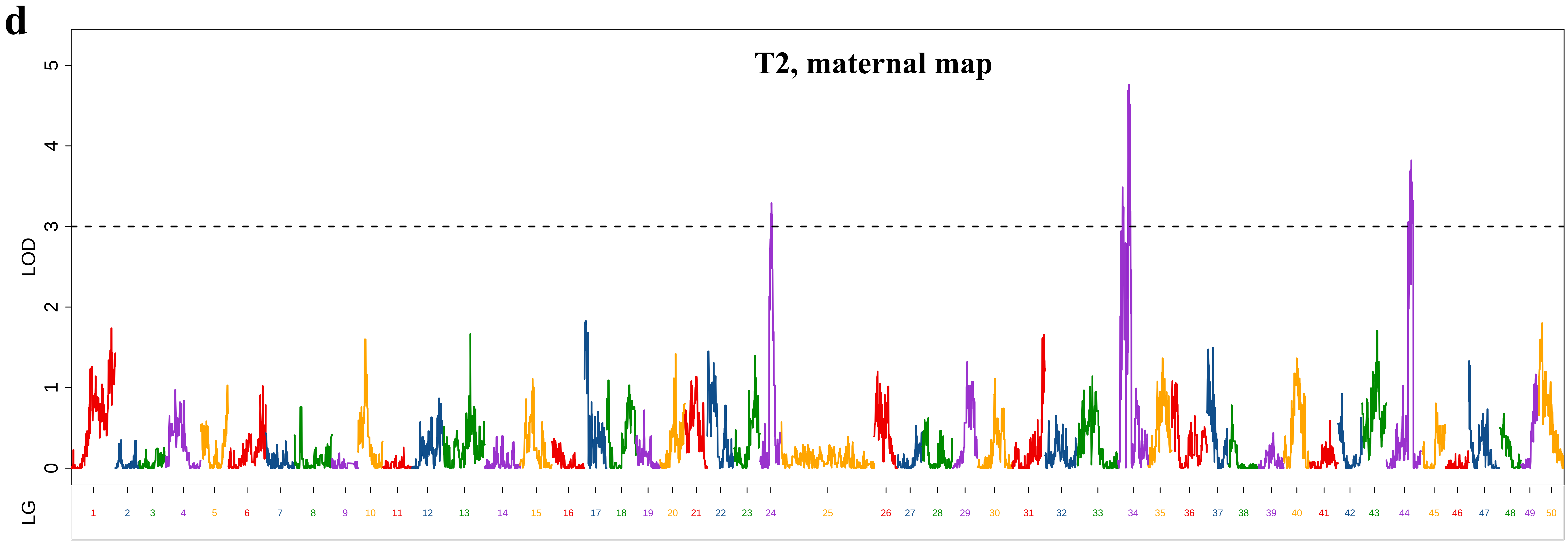
d
T2, maternal map
LOD
0
1
2
3
4
5
LG
1 2 3 4 5 6 7 8 9 10 11 12 13 14 15 16 17 18 19 20 21 22 23 24 25 26 27 28 29 30 31 32 33 34 35 36 37 38 39 40 41 42 43 44 45 46 47 48 49 50

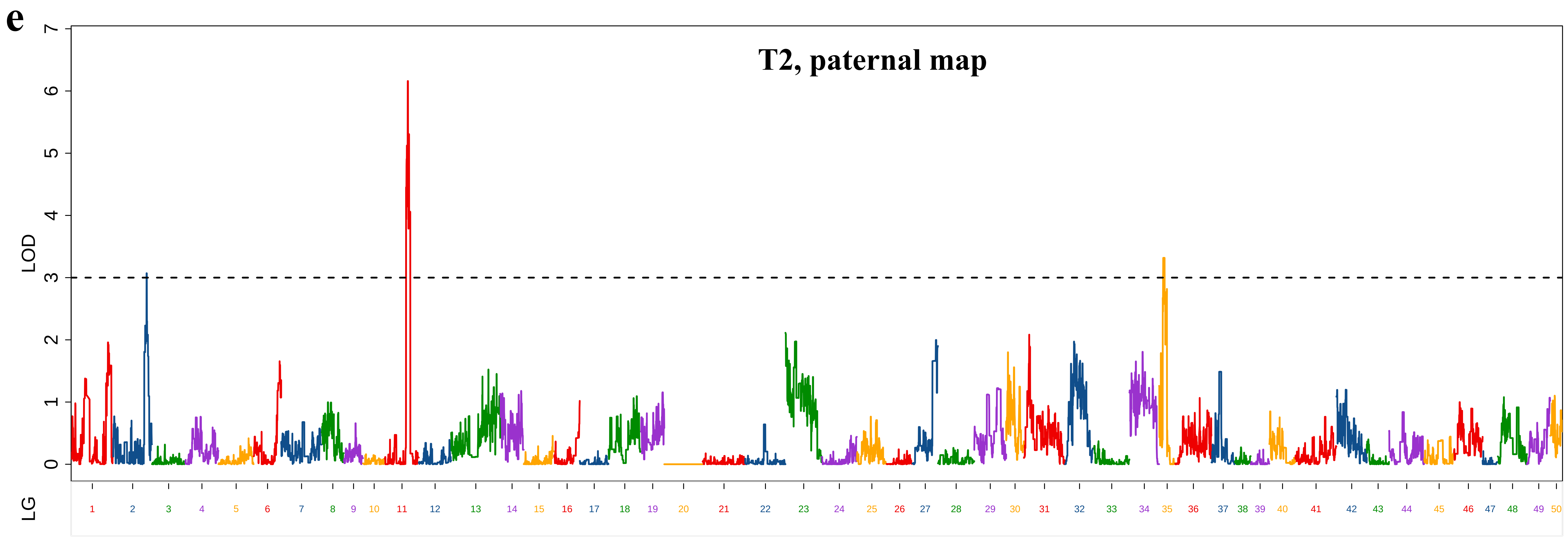
e
T2, paternal map
LOD
0
1
2
3
4
5
6
7
LG
1 2 3 4 5 6 7 8 9 10 11 12 13 14 15 16 17 18 19 20 21 22 23 24 25 26 27 28 29 30 31 32 33 34 35 36 37 38 39 40 41 42 43 44 45 46 47 48 49 50

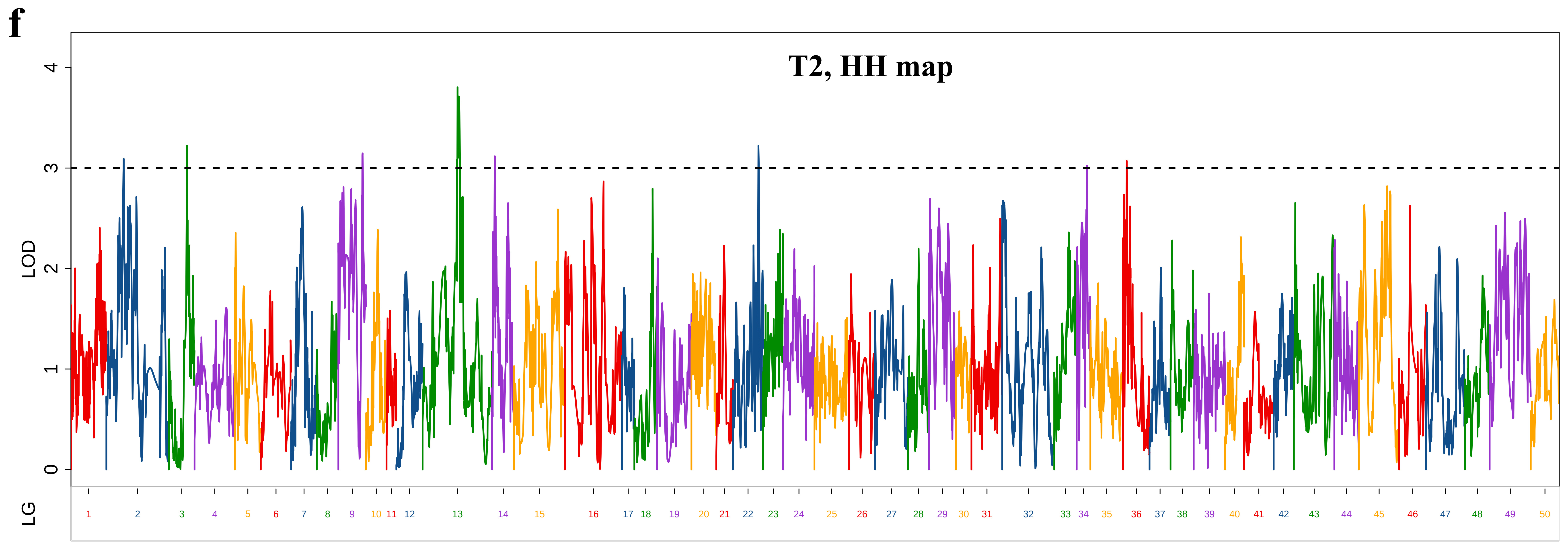
f
T2, HH map
LOD
LG
0
1
2
3
4
1 2 3 4 5 6 7 8 9 10 11 12 13 14 15 16 17 18 19 20 21 22 23 24 25 26 27 28 29 30 31 32 33 34 35 36 37 38 39 40 41 42 43 44 45 46 47 48 49 50

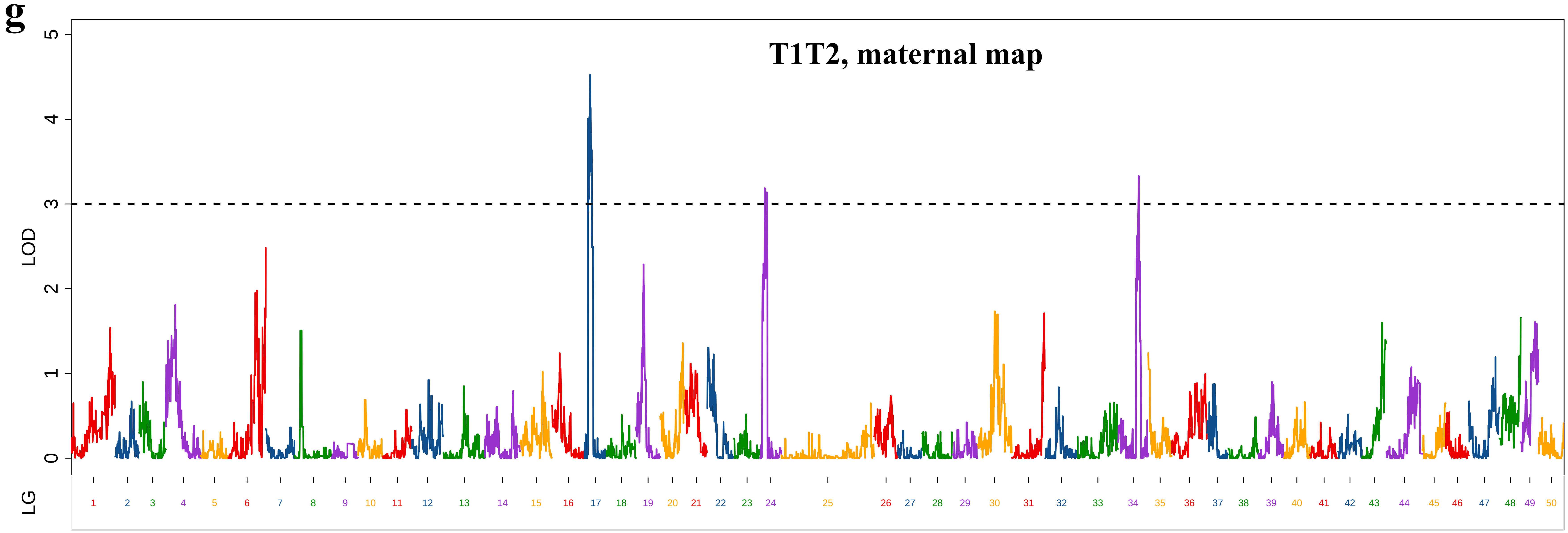

g
T1T2, maternal map
LOD
0
1
2
3
4
5
LG
1 2 3 4 5 6 7 8 9 10 11 12 13 14 15 16 17 18 19 20 21 22 23 24 25 26 27 28 29 30 31 32 33 34 35 36 37 38 39 40 41 42 43 44 45 46 47 48 49 50

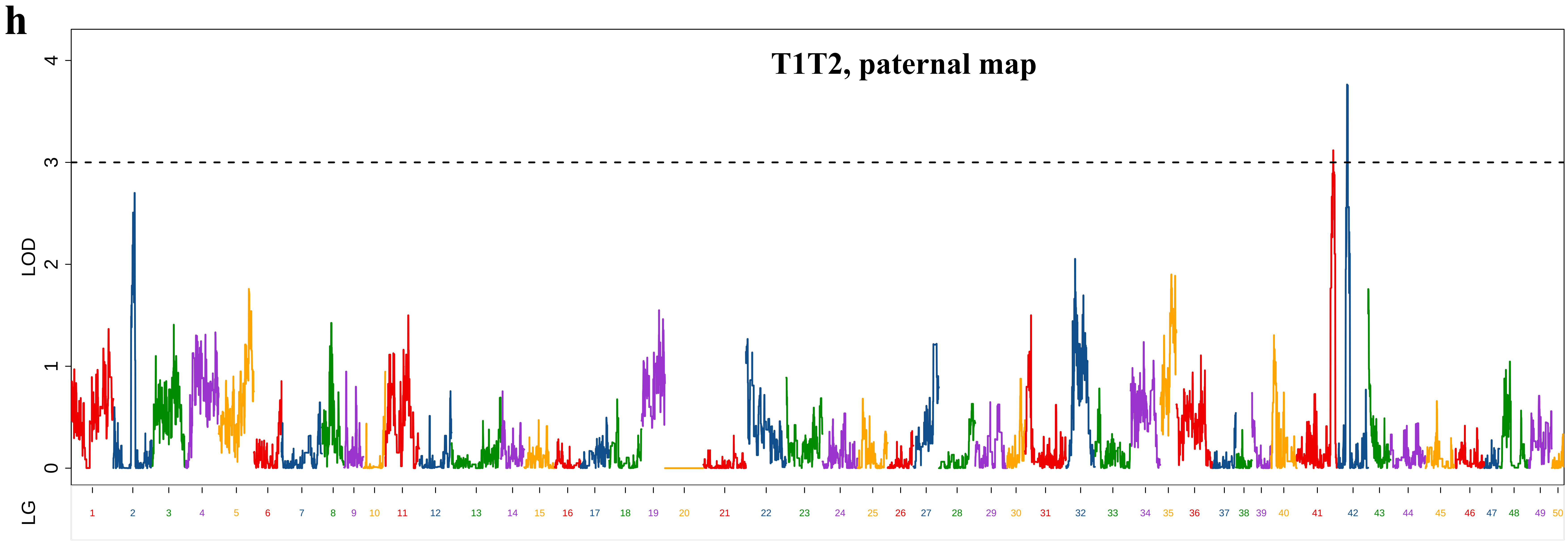
h
T1T2, paternal map
LOD
0
1
2
3
4
LG
1 2 3 4 5 6 7 8 9 10 11 12 13 14 15 16 17 18 19 20 21 22 23 24 25 26 27 28 29 30 31 32 33 34 35 36 37 38 39 40 41 42 43 44 45 46 47 48 49 50

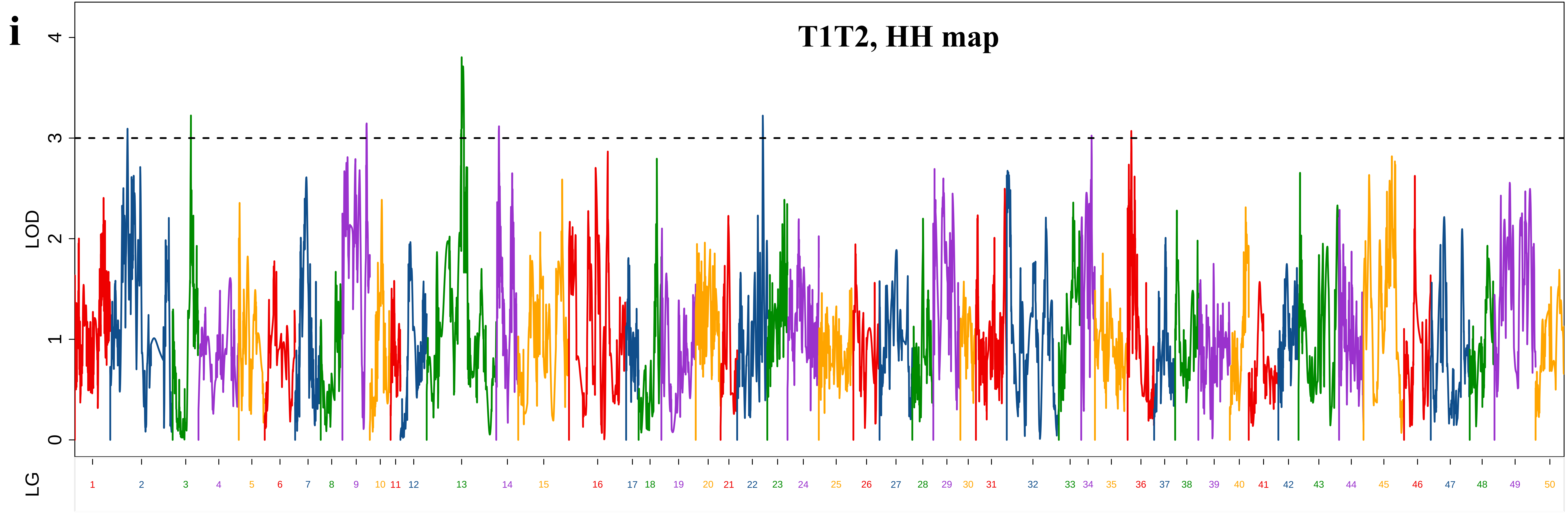

Figure S3. Distribution of body-thickness-related QTLs on 50 linkage groups of Yellow River carp in T1 (a, b, c), T2 (d, e, f) and T1T2 (g, h, i) stages. T1, 13 months old; T2, 17 months old; T1T2, (13-17) months old.
