# Supplementary material for: Construction of Three High-Density Genetic Linkage Maps and Dynamic QTL Mapping of Growth Traits in Yellow River Carp (Cyprinus carpio haematopterus)
Source: Curr Issues Mol Biol. 2021 Dec 17;43(3):2276–88. doi: 10.3390/cimb43030160 (PMC8928983; doi:10.3390/cimb43030160)
Supplement: Supplementary file 1 [file cimb-43-00160-s001.zip › Table S1.pdf]

Table S1. Information about maternal, paternal, and HH genetic linkage maps.

Linkage Group	Maternal map			Paternal map			HH map		
	No. of markers	Length (cM)	Marker intervals (cM)	No. of markers	Length (cM)	Marker intervals (cM)	No. of markers	Length (cM)	Marker intervals (cM)
1	364	179.2	0.49	362	207.5	0.57	401	176.9	0.44
2	346	98.3	0.28	440	191.1	0.43	177	311.8	1.76
3	335	108.7	0.32	431	164.2	0.38	206	129.3	0.63
4	387	141.8	0.37	376	163.6	0.44	109	201.2	1.85
5	361	113.2	0.31	377	173.8	0.46	80	129.8	1.62
6	477	153	0.32	323	136.2	0.42	74	152.6	2.06
7	268	117.7	0.44	409	199.6	0.49	282	128.6	0.46
8	395	153.4	0.39	296	109.3	0.37	74	109	1.47
9	275	106.6	0.39	281	95.6	0.34	89	137.8	1.55
10	342	101.1	0.30	243	108.4	0.45	164	104.6	0.64
11	328	118.5	0.36	308	167.2	0.54	97	50	0.52
12	384	129.7	0.34	275	160	0.58	211	131.2	0.62
13	466	168.2	0.36	626	242.1	0.39	206	347.8	1.69
14	460	144.9	0.32	283	116.2	0.41	192	109.8	0.57
15	327	129.1	0.39	368	150.5	0.41	134	253.7	1.89
16	389	133.7	0.34	334	126	0.38	140	285.3	2.04
17	345	90.2	0.26	302	141.7	0.47	151	62.4	0.41
18	239	118.9	0.50	285	159.9	0.56	105	114.4	1.09
19	369	99.6	0.27	268	115.8	0.43	118	171.7	1.46
20	316	101.9	0.32	352	189.8	0.54	256	124.4	0.49
21	272	91.9	0.34	476	210.1	0.44	76	83.4	1.10
22	319	108.7	0.34	297	199.2	0.67	96	150.5	1.57
23	293	106.3	0.36	356	179.1	0.50	178	101.6	0.57
24	270	88	0.33	426	173.7	0.41	248	156.5	0.63
25	881	377.5	0.43	406	147.5	0.36	234	172.6	0.74
26	293	96.9	0.33	354	126.8	0.36	80	131.3	1.64
27	278	99.6	0.36	241	125.9	0.52	80	163.5	2.04
28	387	123.8	0.32	368	179.6	0.49	147	105.1	0.71
29	221	101.9	0.46	223	157.3	0.71	155	135.8	0.88
30	304	140.5	0.46	275	88.1	0.32	90	78.2	0.87
31	356	136.3	0.38	399	203.8	0.51	246	154.2	0.63
32	422	132.3	0.31	353	142	0.40	193	259.9	1.35
33	438	163.8	0.37	342	176.4	0.52	85	112.7	1.33
34	424	123.9	0.29	364	146.6	0.40	70	68.9	0.98
35	256	95.4	0.37	234	80	0.34	155	163.3	1.05
36	328	143.3	0.44	465	179.7	0.39	184	132.8	0.72
37	370	88.8	0.24	272	114.8	0.42	145	105	0.72
38	317	122.4	0.39	280	78.8	0.28	85	115.7	1.36
39	398	109.4	0.27	251	92.3	0.37	245	157.6	0.64
40	284	106.9	0.38	294	129.1	0.44	141	95.3	0.68
41	282	115.3	0.41	495	201.8	0.41	119	148.6	1.25
42	239	97.5	0.41	400	150.2	0.38	165	100.9	0.61
43	271	100.3	0.37	279	110.6	0.40	125	202.8	1.62
44	308	146.1	0.47	294	172.7	0.59	192	122.7	0.64
45	224	95.2	0.43	241	147.7	0.61	138	202.5	1.47
46	299	95.2	0.32	260	141.2	0.54	92	133.9	1.46
47	342	124.7	0.36	265	75.6	0.29	125	195.4	1.56
48	205	87.8	0.43	258	144.2	0.56	137	124.6	0.91

49	183	72.6	0.40	240	115.1	0.48	90	205.2	2.28
50	249	103.6	0.42	201	61.6	0.31	100	142.1	1.42
Total	16886	6103.6	0.36	16548	7370	0.45	7482	7454.9	1.00
