# Supplementary material for: Construction of Three High-Density Genetic Linkage Maps and Dynamic QTL Mapping of Growth Traits in Yellow River Carp (Cyprinus carpio haematopterus)
Source: Curr Issues Mol Biol. 2021 Dec 17;43(3):2276–88. doi: 10.3390/cimb43030160 (PMC8928983; doi:10.3390/cimb43030160)
Supplement: Supplementary file 1 [file cimb-43-00160-s001.zip › Table S3.pdf]

Table S3. Comparison of genome features for the five Yellow River carp maps.

Parameters	This study	Peng et al. [5]	Wang et al. [4]	Laghari et al. [33]
**Common carp strain**	Yellow River carp	Yellow River carp	Yellow River carp	Common carp
**Number of population**	207	119	108	190
**Mapping family**	F1	F1	F2	F1
**Number of linkage groups**	50/50/50	50	50	50
**Number of linkage markers**	16,886/16,548/7,482	28,194	6,239 SNPs & 65 SSRs	617SSR & 10SNP
**Map length (cM)**	6,103.6/7,370/7,454.9	10,595.94	3,201.90	3,301
**Average length of marker interval**	0.36/0.45/1.00	0.38	0.51	5.60
